# Tumor-mimicking lumbar intradural disc herniation managed with unilateral biportal endoscopy: a case report

**DOI:** 10.1093/jscr/rjag1021

**Published:** 2026-11-05

**Authors:** Bin He, Tao Huang, Haitao Tan, Zhongsen Meng, Bo Sun

**Affiliations:** Key Laboratory of Emergency and Trauma of Ministry of Education, Department of Spine Surgery, The First Affiliated Hospital, Hainan Medical University, Haikou, China; Key Laboratory of Emergency and Trauma of Ministry of Education, Department of Spine Surgery, The First Affiliated Hospital, Hainan Medical University, Haikou, China; Key Laboratory of Emergency and Trauma of Ministry of Education, Department of Spine Surgery, The First Affiliated Hospital, Hainan Medical University, Haikou, China; Key Laboratory of Emergency and Trauma of Ministry of Education, Department of Spine Surgery, The First Affiliated Hospital, Hainan Medical University, Haikou, China; Key Laboratory of Emergency and Trauma of Ministry of Education, Department of Spine Surgery, The First Affiliated Hospital, Hainan Medical University, Haikou, China

**Keywords:** intradural disc herniation, unilateral biportal endoscopy, durotomy, dural patch, nerve sheath tumor

## Abstract

Lumbar intradural disc herniation is rare and frequently mimics intraspinal tumors. A 58-year-old man presented with bilateral L5 radiculopathy and motor weakness. Contrast-enhanced magnetic resonance imaging (MRI) demonstrated an enhancing, cystic L4–5 intraspinal mass favoring a nerve sheath tumor. During unilateral biportal endoscopic surgery, epidural decompression revealed no extradural compression, but persistent thecal tension and absent pulsation prompted an 8-mm dorsal durotomy under reduced irrigation pressure. Sequestered disc fragments adherent to the cauda equina were resected piecemeal, while ventral defect exploration was deliberately avoided. The durotomy was closed using a sutureless inlay dural patch. Histopathology confirmed degenerative disc tissue. The patient achieved complete neurological recovery without cerebrospinal fluid leakage or pseudomeningocele on follow-up MRI. Intradural exploration is warranted when unexplained thecal tension persists, and inlay patch repair serves as an effective endoscopic alternative to primary suturing.

## Introduction

Lumbar intradural disc herniation (IDH) is exceedingly rare, representing ~0.27%–0.3% of all lumbar herniations [1, 2]. Due to inflammatory neovascularization, IDH typically exhibits intense enhancement with cystic changes on contrast-enhanced magnetic resonance imaging (MRI), frequently mimicking schwannomas or other intraspinal tumors [3, 4].

Unilateral biportal endoscopy (UBE) provides continuous fluid irrigation, high-definition visualization, and bimanual instrument manipulation. However, intraoperative decision-making becomes challenging when routine epidural exploration reveals no extradural mass despite prominent MRI findings. Furthermore, performing an intentional durotomy under continuous irrigation raises concerns regarding hydrodynamic intracranial pressure fluctuations, acute neural irritation, postoperative cerebrospinal fluid leakage, and the technical complexity of endoscopic dural repair in a narrow corridor [5, 6].

Herein, we report a case of tumor-mimicking L4–5 IDH successfully treated via UBE, highlighting the intraoperative decision-making for intradural exploration, controlled irrigation techniques, and inlay dural patch reconstruction.

## Case presentation

A 58-year-old man presented with a 2-year history of low back pain and bilateral leg numbness that acutely worsened over 10 days, rendering him unable to walk. Neurological examination showed hypoesthesia in bilateral L5 dermatomes and grade 2/5 muscle strength in bilateral extensor hallucis longus and tibialis anterior (more pronounced on the left); bowel and bladder functions were intact.

Non-contrast lumbar MRI (3 November 2025) demonstrated a well-defined 10 × 8 mm L4–5 intraspinal lesion causing severe thecal compression. Contrast-enhanced MRI revealed marked enhancement with internal cystic degeneration, favoring a presumptive diagnosis of a nerve sheath tumor (Fig. 1).

**Figure 1:**
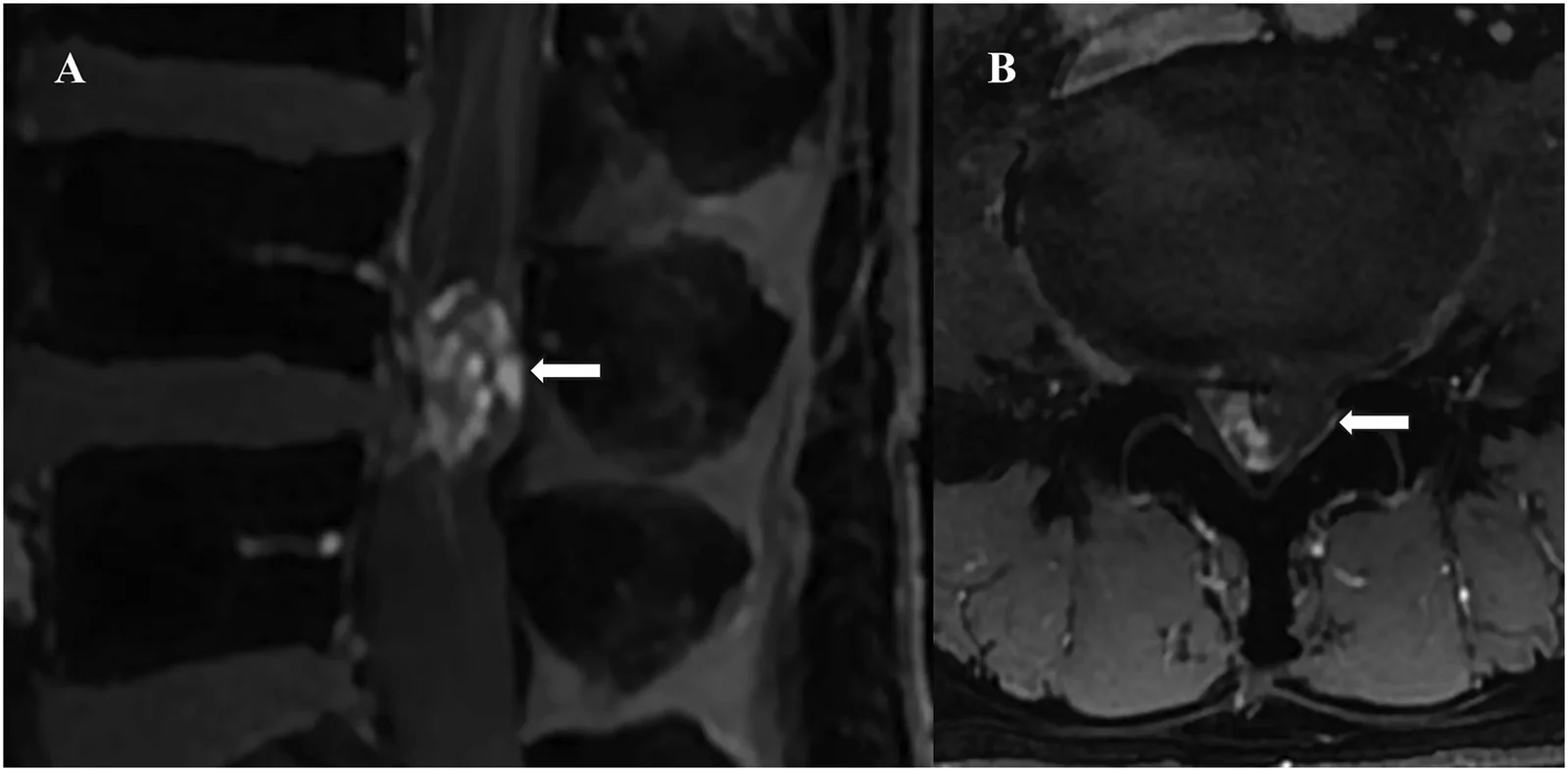
Preoperative contrast-enhanced lumbar MRI at L4–5. (A) Sagittal and (B) axial T1-weighted contrast-enhanced images demonstrate a well-defined, intensely enhancing intracanal mass posterior to the L4–5 interspace, with internal cystic/nonenhancing components and severe dural sac compression (arrows).

A UBE approach was selected for targeted decompression and lesion resection. Following standard laminotomy, flavectomy, and thorough epidural exploration, no compressive extradural pathology was found. Despite adequate canal decompression, the thecal sac remained persistently taut, bulging, and nonpulsatile (Fig. 2A). This discrepancy between preoperative imaging and negative epidural findings indicated an intradural lesion, justifying intradural exploration.

**Figure 2:**
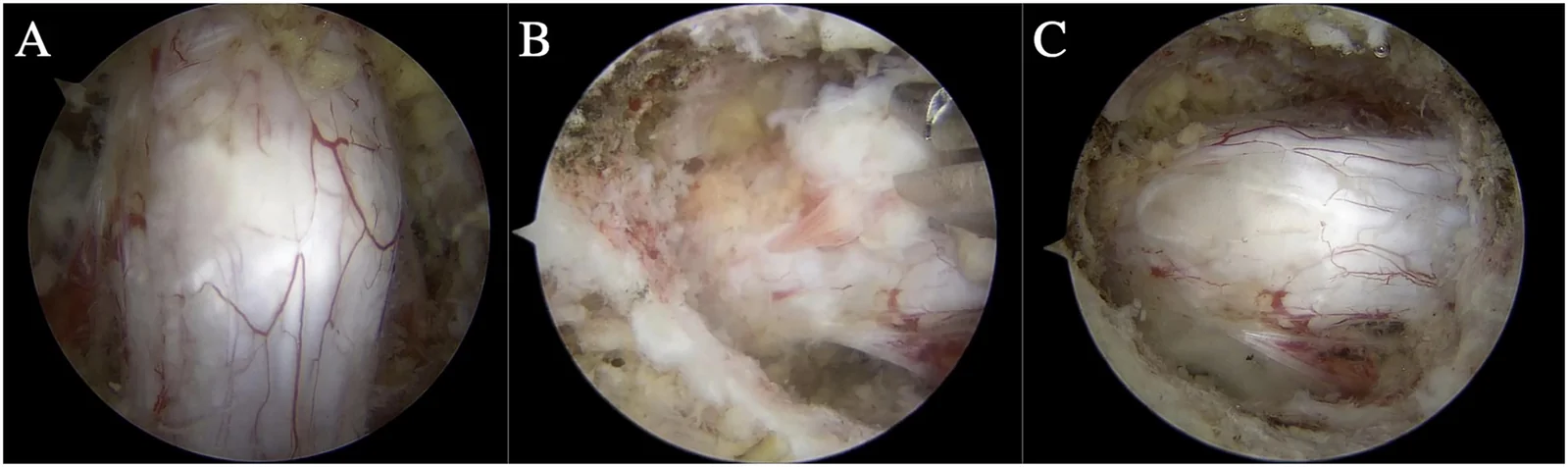
Intraoperative unilateral biportal endoscopic (UBE) views. (A) Persistent thecal tension and absent dural pulsation following thorough epidural decompression. (B) An 8-mm midline dorsal durotomy exposing sequestered fibrocartilaginous disc fragments densely adherent to the cauda equina nerve roots. (C) Inlay repair of the durotomy with an acellular bovine dermal matrix.

Hemostasis was achieved, and inflow pressure was minimized to the lowest level required for optical clarity to prevent fluid overpressure in the subarachnoid space. An 8-mm longitudinal midline dorsal durotomy was performed at the apex of the bulge. Upon opening the arachnoid membrane, firm fibrocartilaginous tissue was identified within the subarachnoid space, densely adherent to the cauda equina (Fig. 2B). The fragments were carefully dissected and removed piecemeal. Ventral defect exploration was deliberately avoided to prevent iatrogenic neural traction. Following lesion evacuation, dural tension resolved immediately, and pulsation was fully restored (Supplementary video 1).

Given the restricted working space and the risk of needle injury to the cauda equina, primary suturing was deemed unfeasible. An acellular bovine dermal matrix was placed as an inlay graft across the durotomy, with its margins gently advanced beneath the dural edges into the subdural space (Fig. 2C). Fibrin sealant was omitted to avoid hydrodynamic washout and potential subarachnoid neurotoxicity. Histopathology confirmed degenerative fibrocartilaginous disc tissue (Fig. 3).

**Figure 3:**
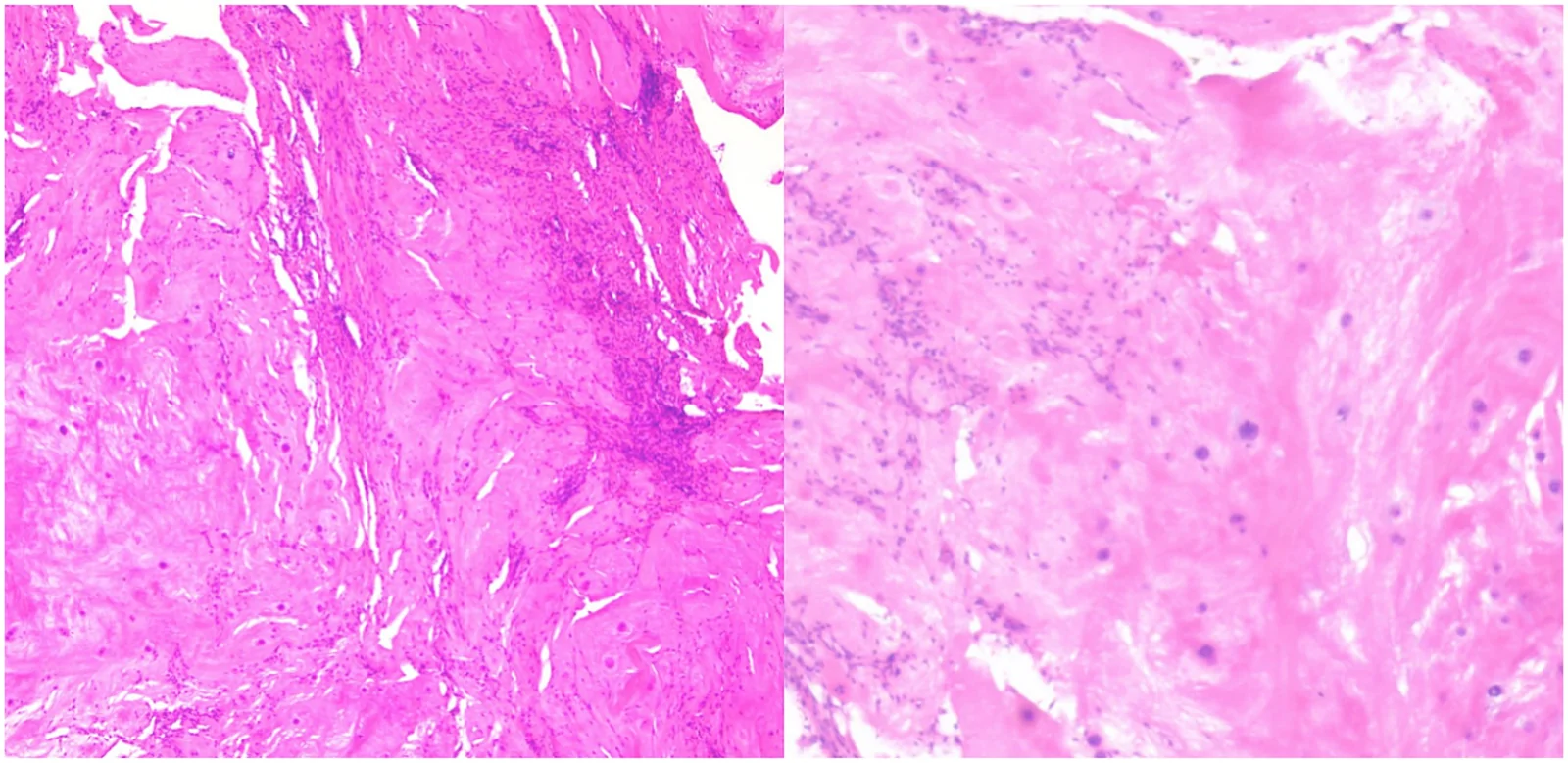
Histopathological evaluation. Hematoxylin and eosin (H&E) staining shows fragmented degenerative fibrocartilage and dense fibrous connective tissue with focal lymphocytic infiltration, consistent with herniated disc tissue (scale bar = 100 μm).

The subfascial drain was removed on postoperative day 3 (<50 ml/day output). The patient experienced no orthostatic headaches, pseudomeningocele, or wound complications. His Japanese Orthopaedic Association score improved from 7/29 preoperatively to 20/29 at 1 week, 27/29 at 1 month, 28/29 at 3 months, and 29/29 at 9 months, with full motor recovery (grade 5/5). Serial follow-up MRIs at 1, 3, and 9 months (26 December 2025, 7 March 2026, and 25 August 2026) confirmed complete decompression without recurrent herniation or pseudomeningocele (Fig. 4).

**Figure 4:**
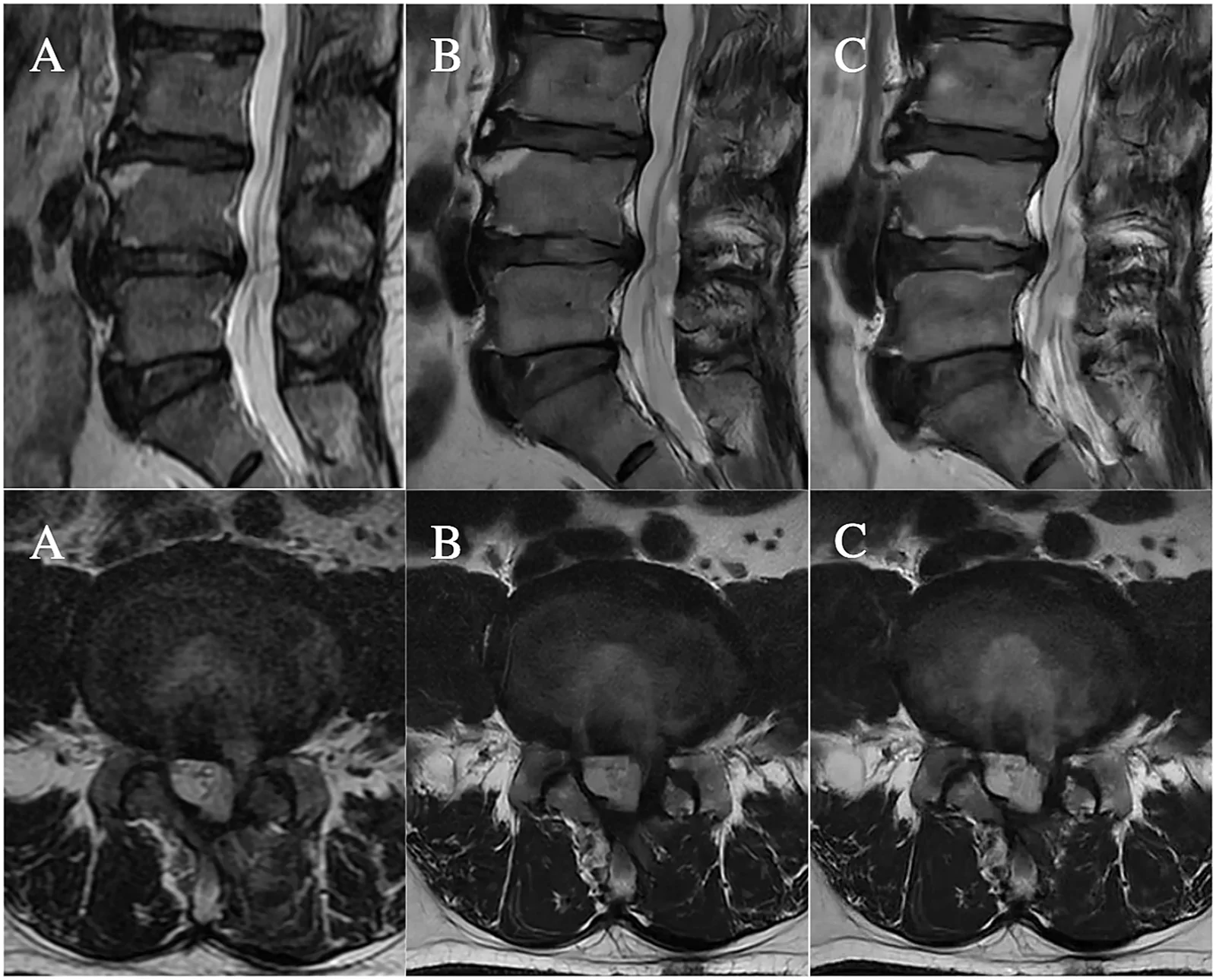
Serial postoperative lumbar MRI. Follow-up sagittal and axial T2-weighted MRI scans obtained at (A) 1 month (26 December 2025), (B) 3 months (7 March 2026) and (C) 9 months (28 August 2026) show sustained decompression of the cauda equina without local fluid accumulation or pseudomeningocele.

## Discussion

The primary diagnostic challenge was the tumor-mimicking radiological appearance combined with negative initial epidural findings. Persistent thecal tension and absent pulsation after complete bony decompression served as a critical intraoperative cue warranting targeted intradural exploration.

Dural reconstruction under continuous irrigation remains challenging in spinal endoscopy. Although watertight primary suturing is standard for dorsal thecal defects [7], needle manipulation in deep endoscopic corridors carries substantial risks of neural impalement. Biportal endoscopic studies demonstrate that small-to-moderate dural defects (<10 mm) can be effectively sealed using patch techniques, muscle grafts, or sealants without open conversion [8, 9]. Kim *et al*. similarly reported successful transforaminal endoscopic excision of lumbar IDH utilizing a dural patch and absorbable gelatin sponge without primary suturing [10].

However, these reports predominantly involve incidental lateral tears or inaccessible ventral defects; thus, sutureless patch repair is not universally equivalent to primary suturing for planned dorsal durotomies. Our strategy was based on risk-benefit analysis: the 8-mm durotomy was small, irrigation pressure was systematically minimized, and the neural elements relaxed postdecompression, whereas forced suturing or ventral defect exploration risked severe cauda equina injury.

Sutureless reconstruction should be applied with extreme caution. Although our patient achieved excellent recovery without pseudomeningocele, this method represents a case-specific rescue strategy rather than a standardized protocol. Limitations include the single-case retrospective design, lack of direct visualization of the ventral defect, and absence of biochemical testing (e.g. beta-2 transferrin) on drainage fluid.

## Supplementary Material

Supplementary_materials_rjag1021
