# Supplementary material for: Tumor-mimicking lumbar intradural disc herniation managed with unilateral biportal endoscopy: a case report
Source: J Surg Case Rep. 2026 Nov 5;2026(11):rjag1021. doi: 10.1093/jscr/rjag1021 (PMC13738226; doi:10.1093/jscr/rjag1021)
Supplement: Supplementary file 1 [file supplementary_materials_rjag1021.zip › Supplementary video 1.docx]

**Supplementary video 1. Surgical video.** Endoscopic dorsal durotomy, piecemeal removal of the intradural mass, and restoration of dural pulsation.
